# Rethinking Japanese Encephalitis Virus Transmission: A Framework for Implicating Host and Vector Species

**DOI:** 10.1371/journal.pntd.0004074

**Published:** 2015-12-10

**Authors:** Jennifer S. Lord, Emily S. Gurley, Juliet R. C. Pulliam

**Affiliations:** 1 Vector Group, Liverpool School of Tropical Medicine, Liverpool, United Kingdom; 2 Department of Biology, University of Florida, Gainesville, Florida, United States of America; 3 Centre for Communicable Diseases, icddr,b, Mohakhali, Dhaka, Bangladesh; 4 Emerging Pathogens Institute, University of Florida, Gainesville, Florida, United States of America; 5 Fogarty International Center, National Institutes of Health, Bethesda, Maryland, United States of America; Centers for Disease Control and Prevention, UNITED STATES

## Current Understanding

Japanese encephalitis virus (JEV) is an important cause of viral encephalitis in Asia, with an estimated 67,900 cases annually [1]. Mosquito-borne zoonoses, including JEV, present some of the most complex disease systems, often involving multiple mosquito and vertebrate species.

**Fig 1:**
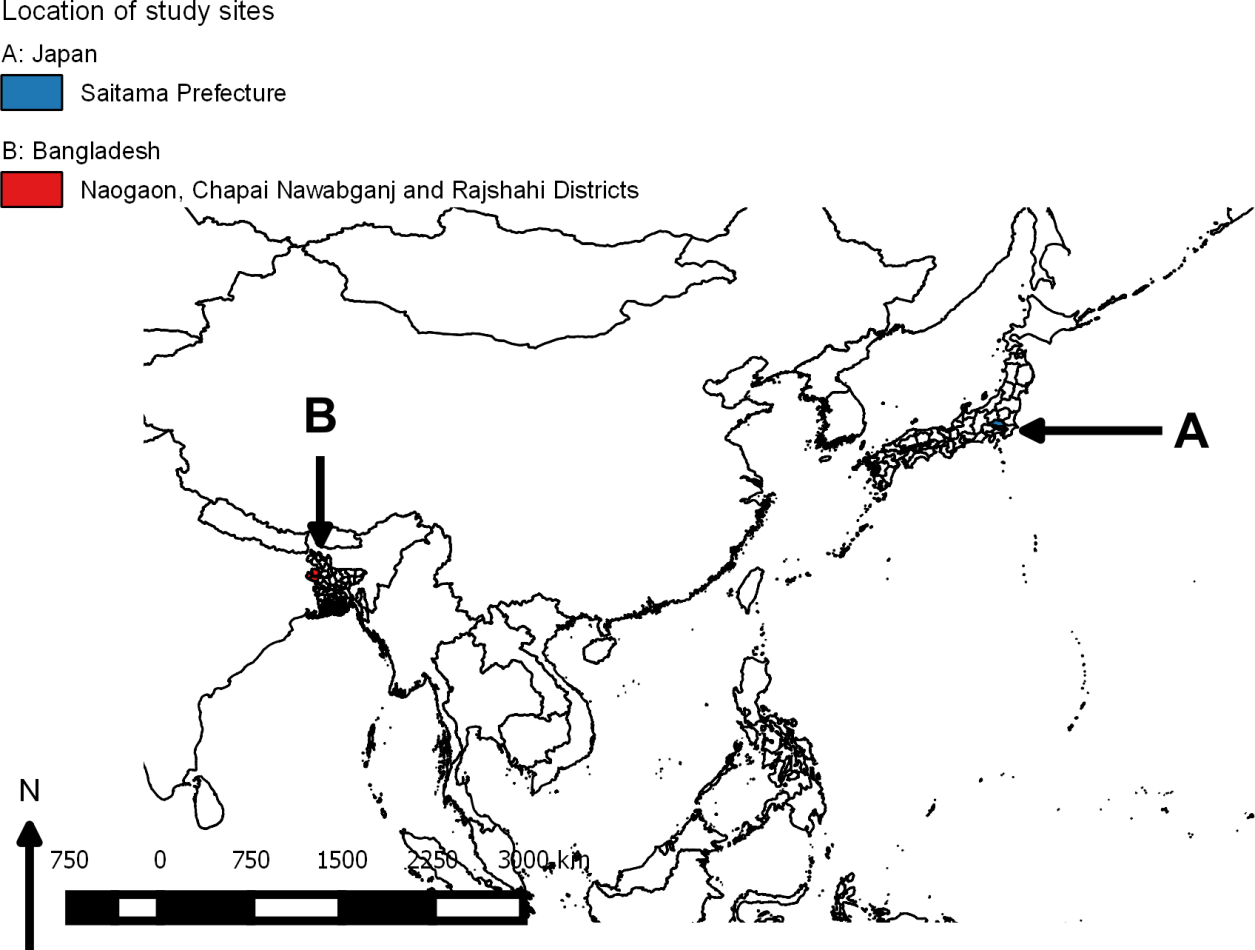
Study locations in Japan (A) and Bangladesh (B) where host community composition has been estimated (Fig 2).

The first investigations of JEV transmission ecology were undertaken in the 1950s in Saitama Prefecture, Japan (Fig 1A) [2–10]. As a result of these studies, *Culex tritaeniorhynchus* was implicated as the primary vector and pigs as the amplifying hosts, with a minor role described for ardeid birds [10]. Scherer et al. [3] justified the intensive investigation of pigs and birds in Japan by emphasizing that, within this context, only these animals and wild rodents underwent population turnover high enough to provide the continuous supply of susceptible individuals necessary to explain the occurrence of annual epidemics. Research was focused on these species, in preference to other animals, including cattle, whose total and susceptible populations were smaller. Among potential bird hosts, ardeid birds in particular were studied because they possessed anti-JEV antibodies, were numerous, colonial, could be caught in large numbers, and were large enough to withstand repeated bleedings adequate for testing. Their selection was not meant to imply that other birds were not potentially important in JEV ecology [3].

**Fig 2:**
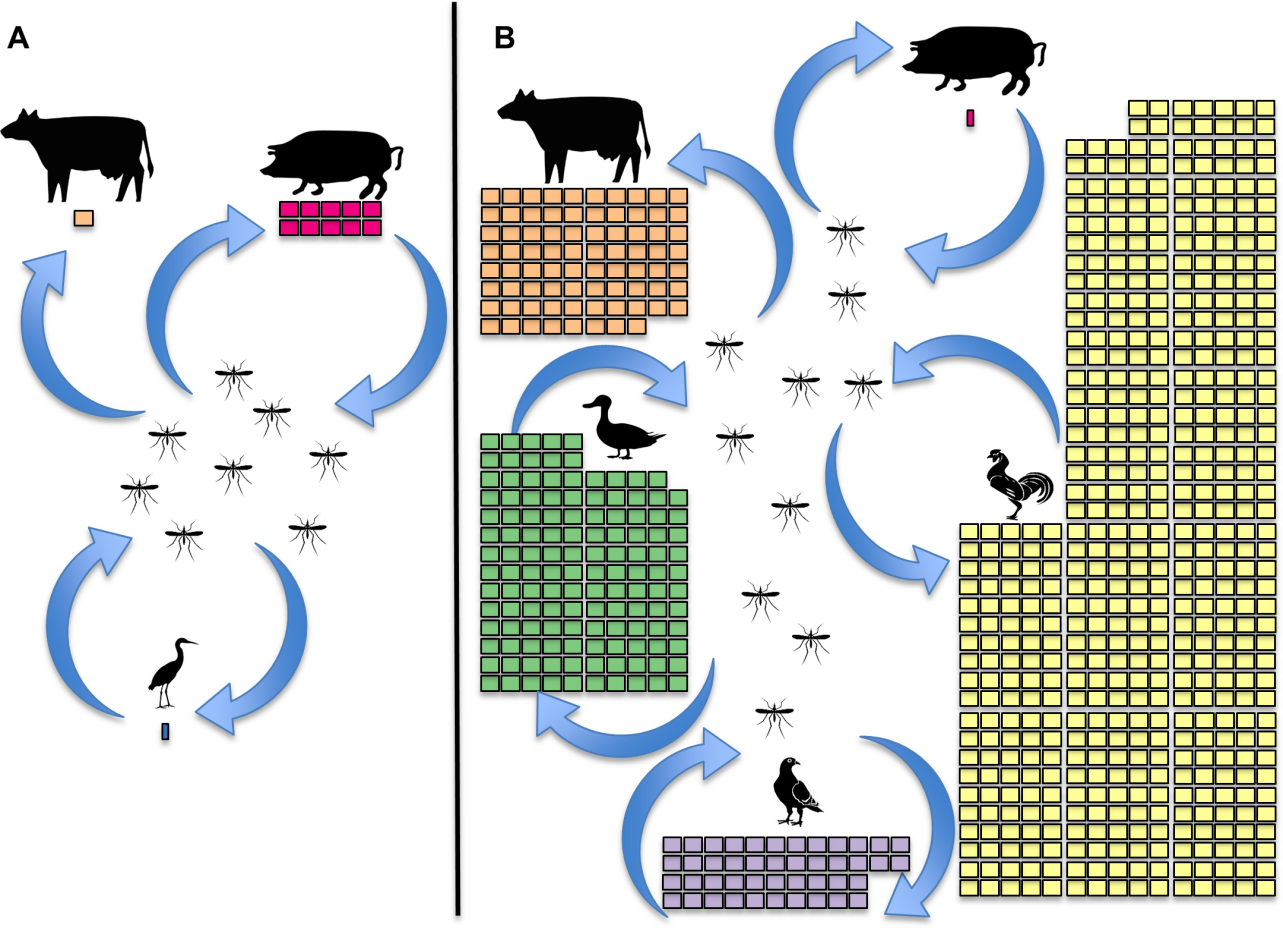
Comparison of JEV transmission contexts between Saitama Prefecture, Japan (A), and three districts of Bangladesh (Rajshahi, Naogaon, and Chapai Nawabganj) (B), with respect to host community composition. Arrows represent hypothesized transmission of JEV between hosts and mosquitoes. Each square represents approximately 10,000 animals; smaller squares for pigs in Bangladesh and ardeid birds in Japan represent proportionately smaller numbers. Saitama Prefecture covers approximately 3,800 km^2^ and the three districts of Bangladesh approximately 7,500 km^2^. Data for Bangladesh were, therefore, scaled so that densities between the two regions are comparable. Saitama Prefecture census data for cattle and pigs were taken from [8], field estimates of ardeid birds from [7]. Bangladesh data for pigs were from [20], and cattle, pigeons, ducks, and chickens from Bangladesh Yearbook of Agricultural Statistics [19]. As ducks and chickens were reported together, an approximate ratio of 1:5 was calculated from FAOSTAT for 2012 [21]. Cattle and bird absolute numbers and density in Bangladesh are much higher than was observed in Japan. Given the context in Bangladesh, it is not currently fully understood how JEV transmission is maintained, and we propose that domesticated birds may play an important role.

The transmission cycle proposed from this initial research in Japan arose from careful study of the transmission context in that location, at that time. Vertebrate population density (Fig 2), life span, and JEV viremia were considered when implicating primary hosts [6–8]. Baited mosquito traps were used to determine numbers of mosquitoes attracted to a variety of bird species, pigs, and humans [5]. The relative abundance of each mosquito species caught in baited traps and their JEV infection status were compared when implicating vectors in transmission [4]. *Cx*. *tritaeniorhynchus* was found to be most abundant in traps baited with hosts able to produce JEV viremia [2,4], providing circumstantial evidence for this species’ role in transmission, which was strengthened by laboratory experiments demonstrating this mosquito’s competence for JEV replication and transmission [2].

As highlighted by numerous review articles, the initial investigations in Japan have formed the basis for describing the JEV transmission cycle, primarily involving *Cx*. *tritaeniorhynchus*, pigs, and, to a lesser extent, ardeid birds [11–15].

## Considering Transmission Context

The *Cx*. *tritaeniorhynchus*–pig transmission cycle first described in Japan occurred in a context where pigs were intensively farmed and were the most numerous of possible, competent, vertebrate hosts. Yet not all regions of Asia experiencing Japanese encephalitis (JE) outbreaks reflect this scenario.

JE cases do occur in the absence of intensive pig farming and where pig density is low relative to other livestock, including in regions of Bangladesh and India [1]. Unlike Japan, in Bangladesh, Islam is the largest religion. As a consequence, pig farming can be associated with social stigma in this region, thus restricting its growth as an industry [16].

Pig density relative to cattle density is particularly important to consider in JEV transmission ecology. Cattle are unable to produce viremia sufficient to infect mosquitoes under experimental conditions and, thus, are a “dead end” for JEV [17]. During outbreaks of JE in the 1950s in Saitama Prefecture, Japan (Fig 1A), there was a high pig population turnover, with approximately 100,000 pigs slaughtered annually, and pig densities were reported to be ten times higher than cattle (Fig 2A) [8]. In contrast, in some JE-endemic regions of India, cattle can outnumber pigs by up to 20:1 [18]. In three JE-endemic districts of Rajshahi Division, Bangladesh (Fig 1B), which, together, cover an area almost twice the size of Saitama, the pig population is estimated to be 11,000 and the cattle population over 1 million—140 cattle for every pig (Fig 2B) [19,20].

When given a choice between feeding on a cow or a pig under experimental conditions, 42% of 496 *Cx*. *tritaeniorhynchus* fed on the cow and 5% on the pig [22]. Blood feeding of natural populations of *Cx*. *tritaeniorhynchus* in India has been observed to be between 85% and 98% on cattle and less than 10% on pigs [18,23,24]. This compares with 36% on cattle and 55% on pigs in Japan [25]. These differences are likely due to differences in the availability of the respective hosts. Theoretical models of vector-borne pathogen transmission [26] demonstrate that the rate of pathogen spread is particularly sensitive to the proportion of vector bloodmeals taken from competent versus dead-end hosts. This is because the proportion of bloodmeals taken on each host species influences both mosquito-to-host and host-to-mosquito transmission rates, forming a squared term in an equation for the basic reproduction number of a vector-borne pathogen. If mammalophilic vectors such as *Cx*. *tritaeniorhynchus* are more likely to feed on cattle than pigs, transmission intensity may decrease if cattle density substantially exceeds pig density [27]. While it is possible there are sufficient mosquitoes per host in tropical regions for pigs to maintain transmission irrespective of the proportion of bites on pigs, the size of the reservoir community required for JEV amplification to levels that are a risk to human populations is unknown. Are pig population densities in regions of India and Bangladesh sufficient for maintaining JEV transmission? Our understanding of the drivers of JEV transmission in regions that differ in transmission context from Japan is currently deficient.

In light of the potential expansion of JEV to new geographic regions that support a range of livestock and agricultural practices (http://faostat.fao.org/) [14,28,29], it is important that the transmission cycle be reconsidered for regions of Asia where the transmission context may differ substantially from that first described in Japan (Fig 2).

## Reassessing the JEV Transmission Cycle

During entomological investigations in ten randomly selected villages in a JE-endemic region of Bangladesh [30], birds—including chickens, ducks, and pigeons—were observed to be the most abundant domestic animals, often comprising between 50% and 100% of household animal communities. These birds are also reported to be the most numerous domestic animals by census data for the three districts where surveyed villages are located [19].

In addition to density, the amount of virus present in host blood after a bite by an infectious mosquito is also an important parameter in determining the extent to which a host may contribute to transmission [31]. Viremia profiles of pigeons, ducks, chickens, and pigs have, to our knowledge, yet to be compared with respect to the probability of mosquito infection; however, experimental infection studies for these animals are available [2,8,32–35]. Whilst the amount and strain of JEV administered likely differ between studies, the amount of virus detected in an individual host on any day post-infection was similar between species. Pig viremia has been recorded to vary between 0.4 and 3.3 log_10_ lethal dose (LD) _50_ / 0.03 ml, compared with 0.2 to 1.7 for pigeons, 0.5 to 3.4 for chickens, and 0.6 to 4.5 for ducks [2,8,32–34]. Although ducks and chickens are, therefore, likely to produce JEV viremia sufficient to infect mosquitoes [2,8, 31,32–35], the role of domesticated birds in JEV transmission remains unknown. The involvement of ducks in JEV transmission, in particular, was suggested as a possibility in Borneo, but their contribution to transmission there also remains to be quantified [36]. Quantifying the relative contributions of pigs and domesticated birds to JEV transmission is essential for understanding JEV ecology in regions where the pig population density is relatively low compared with the domesticated bird population density (Fig 2B). We propose that several competing hypotheses should be evaluated: (i) pigs contribute more than domesticated birds to JEV transmission; (ii) domesticated birds contribute more than pigs to JEV transmission; (iii) the relative contributions of domesticated birds and pigs varies in space and time. There are, however, currently insufficient data to fully assess these hypotheses.

Efforts to accurately quantify the contribution of different hosts and vectors to JEV transmission are hindered by the need to simultaneously assess multiple parameters [26]. These parameters include population density of multiple species, mosquito species’ blood feeding habits, and the ability of species to become infected and subsequently transmit JEV. As applied to the study of West Nile virus in the USA [37], the use of mathematical models parameterized with data from entomological and host-based studies would be useful in quantifying the relative roles of potential species in JEV transmission, but this approach has not, thus far, been applied to the JEV system, in part due to inadequate data.

Estimation of the parameters necessary for implicating host and vector species may be affected by method bias (for example, mosquito collection methods that favor one species over another) [37], and parameter estimates may differ across scales, space, and time due to ecological heterogeneity (Table 1). These factors are important to consider, as bias and heterogeneity may influence parameters for each species under consideration in different ways that would need to be accounted for when using mathematical models (Table 1). In addition, many mosquito species can become infected and, subsequently, transmit JEV. Further investigations—including bloodmeal analyses, use of mosquito sampling methods that focus collections on competent rather than dead-end host species present in an area, and JEV competence experiments—would improve our understanding of the host and vector species driving JEV transmission.

**Table 1:** Summary of potential sources of bias and heterogeneity that may influence estimation of parameters used to implicate host and vector species in Japanese encephalitis virus transmission.

Parameter	Data source	Bias/ heterogeneity	Potential implications	Recommendation
**Mosquito species relative abundance**	Often estimated from sampling near large domestic animals, particularly cattle, at dusk [30].	Over-representation of dusk-biting and/or mammalophilic species, including the *Cx*. *vishnui* subgroup (*Cx*. *tritaeniorhynchus*, *Cx*. *vishnui*, *Cx*. *pseudovishnui*) in the studied mosquito community relative to other species and under-representation of day-biting and/or ornithophilic species [30].	May reinforce current theory of a *Cx*. *tritaeniorhynchus*–pig cycle, creating a barrier to recognition of alternative transmission cycles.	Use a combination of methods. These may include: collections focused near hosts known to produce JEV viremia both during the day and in the evening; collections of resting mosquitoes away from potential host animals, indoors as well as outdoors.
**Host and mosquito species competence (ability to become infected and subsequently transmit a pathogen)**	Estimated from experimental laboratory transmission experiments.	Usually taken to be two constant parameters that are not influenced by environmental factors. Mosquito competence is, however, affected by host viremia (aspect of host competence), and this relationship may be temperature-dependent [38].	Assuming constant host-to-mosquito and mosquito-to-host transmission probabilities may lead to failure to account for regional differences in host and vector species competence due to environmental conditions.	Experimental infections should be conducted to quantify how the probability of mosquito midgut and salivary gland infection varies with dose and temperature. Such experiments will give insight into the relationships between environmental factors and transmission probabilities.
**Mosquito species’ host-feeding patterns**	Usually averaged over a region including multiple villages [18,23,39].	May not account for poor mixing between host species and vectors across spatial scales [40].	May overestimate the proportion of bloodmeals taken on dead-end rather than competent species in an area, resulting in failure to understand how transmission is maintained.	Identification of the scale at which host community composition varies. Quantification of the proportion of bloodmeals on each host species at this scale (for example, at the household rather than village level).

## Implications for Control

Quantifying the relative contributions of species involved in JEV transmission, and the role of birds in particular, would improve assessments of both the potential for JEV to spread to new geographic regions [14,28,29] and the potential impact of particular farming systems, including duck farming in rice paddies [41].

Japanese encephalitis is a vaccine-preventable disease and has been successfully controlled by national human immunization programs in Japan, Taiwan, China, and Korea [1]; however, the disease is still a major public health problem in many regions of Asia, including Bangladesh and India [1]. The cost of national immunization programs and the logistics of vaccinating all individuals in at-risk areas currently restrict use in some JE-endemic regions [42]. Furthermore, as human infection does not contribute to transmission and the human vaccine does not reduce transmission of JEV in the reservoir community, no herd immunity is generated, and vaccination has to be sustained indefinitely. Implicating host and vector species would improve understanding of transmission risk in space and time, and could, therefore, inform targeted vaccination efforts toward those at highest risk.
